# Radiomic Machine-Learning Analysis of Multiparametric Magnetic Resonance Imaging in the Diagnosis of Clinically Significant Prostate Cancer: New Combination of Textural and Clinical Features

**DOI:** 10.3390/curroncol30020157

**Published:** 2023-02-07

**Authors:** Francesco Prata, Umberto Anceschi, Ermanno Cordelli, Eliodoro Faiella, Angelo Civitella, Piergiorgio Tuzzolo, Andrea Iannuzzi, Alberto Ragusa, Francesco Esperto, Salvatore Mario Prata, Rosa Sicilia, Giovanni Muto, Rosario Francesco Grasso, Roberto Mario Scarpa, Paolo Soda, Giuseppe Simone, Rocco Papalia

**Affiliations:** 1 Department of Urology, Fondazione Policlinico Universitario Campus Bio-Medico, 00128 Rome, Italy; 2 Department of Urology, IRCCS “Regina Elena” National Cancer Institute, 00144 Rome, Italy; 3 Unit of Computer Systems and Bioinformatics, Department of Engineering, Fondazione Policlinico Universitario Campus Bio-Medico, 00128 Rome, Italy; 4 Department of Diagnostic and Interventional Radiology, Sant’Anna Hospital, 22042 San Fermo della Battaglia, Italy; 5 Simple Operating Unit of Lower Urinary Tract Surgery, SS. Trinità Hospital, 03039 Sora, Italy; 6 Department of Urology, Humanitas Gradenigo University, 10153 Turin, Italy; 7 Department of Diagnostic and Interventional Radiology, Fondazione Policlinico Universitario Campus Bio-Medico, 00128 Rome, Italy

**Keywords:** clinically significant, machine-learning, prostate biopsy, prostate cancer, radiomic

## Abstract

Background: The aim of our study was to develop a radiomic tool for the prediction of clinically significant prostate cancer. Methods: From September 2020 to December 2021, 91 patients who underwent magnetic resonance imaging prostate fusion biopsy at our institution were selected. Prostate cancer aggressiveness was assessed by combining the three orthogonal planes-Llocal binary pattern the 3Dgray level co-occurrence matrix, and other first order statistical features with clinical (semantic) features. The 487 features were used to predict whether the Gleason score was clinically significant (≥7) in the final pathology. A feature selection algorithm was used to determine the most predictive features, and at the end of the process, nine features were chosen through a 10-fold cross validation. Results: The feature analysis revealed a detection accuracy of 83.5%, with a clinically significant precision of 84.4% and a clinically significant sensitivity of 91.5%. The resulting area under the curve was 80.4%. Conclusions: Radiomic analysis allowed us to develop a tool that was able to predict a Gleason score of ≥7. This new tool may improve the detection rate of clinically significant prostate cancer and overcome the limitations of the subjective interpretation of magnetic resonance imaging, reducing the number of useless biopsies.

## 1. Introduction

According to American Cancer Society data, prostate cancer (PCa) is the most common cancer and the second leading cause of deaths in male individuals. European Association of Urology (EAU) guidelines suggest offering PSA testing in men after 50 years of age, or earlier if risk factors are known [1]. Recent studies outlined the relativity of PSA test levels [2]. Furthermore, PSA screening has resulted in little or no reduction in PCA-specific mortality, while it may be responsible for unnecessary treatments following overdiagnosis [3]. Magnetic resonance imaging (MRI) has increasingly been used for PCa diagnosis [4,5]. EAU guidelines strongly recommend performing multiparametric MRI (mpMRI) before prostate biopsy in both naïve and priorly negative biopsy patients, adhering to the Prostate Imaging Reporting and Data System (PI-RADS) guidelines for interpretation [1]. Several PI-RADS versions have been adopted to try to overcome the limitations of the system [4,6,7]. Clinically significant (CS) PCa, according to PI-RADS v2, is defined as a PCa with a histopathology ISUP grade of ≥2 and/or volume of ≥0.5 cc and/or with extraprostatic extension [8]. However, there are lesions with questionable probability of being considered part of clinically significant (CS) PCa, and this still represents a clinical challenge: up to 20–30% of them will turn out to be malignant [4]. The radiomic-based machine learning tools have shown to potentially increase the predictive efficiency of PI-RADS [9]. By extracting quantitative data from radiological images, radiomics can build computational models to predict the presence of cancer. The aim of our study was to develop a non-invasive radiomics tool by using T2w and apparent diffusion coefficient (ADC) MRI images, addressing its accuracy, sensitivity, and specificity for the prediction of CS PCa.

## 2. Materials and Methods

### 2.1. Patient Cohort

From September 2020 to December 2021, we screened all biopsy-naïve patients who underwent prostate mpMRIs and prostate biopsies at our Institution from September 2019 to December 2020 with potential PCa due to an elevated PSA and/or a positive digital rectal examination (DRE). The 135 patients selected underwent trans-rectal ultrasound-guided (TRUS) MRI target fusion biopsies plus 12-core systematic biopsies, according to EAU clinical guidelines [1]. All biopsies were performed within a month from the mpMRIs.

Patients were included in our study according to the following criteria:

- Serum PSA ≤ 20 ng/mL;
- Patients who underwent mpMRI and fusion prostate biopsy plus standard 12-core systematic TRUS biopsy within a month from the MRI;
- Suspicious prostate lesion areas (PIRADS ≥ 3);
- Prostate lesions with definite boundaries (regular margins) and classified according to the latest version of PI-RADS score [9];
- Clinical stage ≤ T2 at mpMRI (organ-confined PCa).
- Exclusion criteria were defined as follows:
- Prior treatment for PCa;
- Poor MRI image quality due to serious image artefacts;

Precluded segmentation due to the size and/or location (most basal and apical MRI slices) of cancer lesions.

### 2.2. MRI Image Acquisition and Region of Interest Delineation

All MRIs were performed on a 1.5 T MRI system (Magnetom Aera Siemens Erlangen^®^, Erlangen, Germany). An endorectal coil was not used, and a dedicated six-channel body coil was positioned over the pelvis with the patient in the supine position. After localizer sequences were taken in three orthogonal planes, the following protocol was adopted:

- Sagittal T2-weighted turbo spin echo (slice thickness TR 4000.0 ms, 3.0 mm; TE 114.0 ms; Voxel size 0.7 × 0.7 × 3 mm; field of view (FoV) 180 × 180 mm; nex 2; concatenation 2);
- Coronal T2-weighted turbo spin echo (slice thickness TR 4400.0 ms, 3.0 mm; TE 114.0 ms; voxel size 0.7 × 0.7 × 3 mm; field of view (FoV) 180 × 180 mm; nex 2; concatenation 2);
- Axial T2- weighted turbo spin echo (lice thickness TR 4000.0 ms, 3.0 mm; TE 114.0 ms; voxel size 0.7 × 0.7 × 3 mm; field of view (FoV) 180 × 180 mm; nex 2; concatenation 2);
- Axial T1-weighted turbo spin echo (slice thickness TR 568.0 ms, 3.0 mm; TE 11.0 ms; voxel size 0.8 × 0.8 × 3 mm; field of view (FoV) 200 × 200 mm; nex 2; concatenation 2);
- Axial T2-weighted spectral attenuated inversion recovery (slice thickness TR 5310.0 ms, 3.0 mm; TE 95.0 ms; voxel size 0.8 × 0.8 × 3 mm; field of view (FoV) 200 × 200 mm; nex 2; concatenation 2);
- Axial single-shot echo-planar (SSEP) diffusion-weighted sequence with diffusion-sensitizing gradient applied along the *x*, *y*, *z* axes and with a b value of 50, 500, 800 and 1000 s/mm^2^ (slice thickness TR 4300.0 ms, 4.0 mm; TE 73.0 ms; voxel size 0.9 × 0.9 × 0.9 mm; field of view (FoV) 240 × 240 mm; concatenation 1);
- Axial T1-weighted dynamic volumetric interpolated breath-hold examination (VIBE) fat suppressed sequence (slice thickness TR 4.46 ms, 4.0 mm; TE 1.63 ms; voxel size 1.2 × 1.2 × 4 mm; field of view (FoV) 260 × 260 mm; nex 1; concatenation 1). The contrast agent, gadobenate-dimeglumine (Multihance^®^, Bracco Imaging, Milan, Italy), was administered in a concentration of 0.2 mmol/kg; it was injected with an automatic injector through a 20 G intravenous cannula at the rate of 4 mL/s, followed by the infusion of 15 mL of saline solution at the same speed. The contrast agent and the sequence started simultaneously to assess the perfusion of the organ. The sequence was acquired once before and 18 times after the contrast injection (echo trains) for a total duration of 3.7 ± 0.5 min. Subtracted images were automatically derived from DCE-MRI.
- Axial T1-weighted VIBE fat suppressed sequence (slice thickness TR 4.76 ms, 2.0 mm; TE 1.82 ms; oxel size 1 × 1 × 1 mm; field of fiew (FoV) 200 × 200 mm; nex 2; concatenation 1).

An experienced radiologist in urogenital imaging (with 12 years of experience and more than 1000 mpMRIs reviewed) evaluated and reviewed the mpMRIs of patients included, identifying lesions with suspectedr PCa. All lesions were classified according to the latest PI-RADS score system [10].

**Figure 1:**
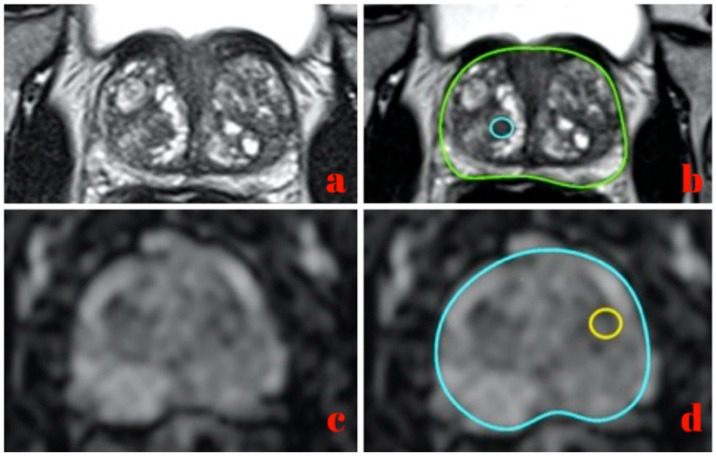
Image segmentation on a T2w MRI slice (**a**,**b**) and ADC MRI slice (**c**,**d**).

The radiologist manually delineated all the lesions using a dedicated software (OsiriX DICOM viewer software, version 10.0.4),drew a circular region for each slice in which the prostate lesion was detectable, and refined the margins according to the lesion’s shape if necessary (Figure 1), generating a volume of interest (VOI). ROIs were independently drawn for T2w or ADC based on where the radiologist pointed out the lesions.

### 2.3. Fusion Biopsy and Pathological Examination

Prostate biopsies were carried out as outpatient procedures using the Koelis UroStation^®^ platform (Koelis, La Tronche, France) equipped with an external ultrasound system (Samsung H60, Samsung Healthcare^®^, Seoul, Republic of Korea) with a 4–9 MHz 3D TRUS probe (Samsung 3D4-9 3D endocavitary probe). Povidone-iodine rectal preparation before biopsies was performed. Antibiotic prophylaxis consisted of the peri-procedural intramuscular injection of 1.5 mg/kg of aminoglycoside. Trans-rectal peri-prostatic anaesthetic block was performed using a 22-G Chiba needle (RONN2222ST, ROCAMED^®^) with 5 mL of Lidocaine 20 mg/mL plus 5 mL of Bupivacaine 5 mg/mL. Biopsies were executed trans-rectally with an 18-G biopsy needle biopsy gun (MC1825, BARD MAX-CORE^®^). MRI target TRUS fusion biopsies plus standard 12-core systematic biopsies (six cores for each lobe from the base to the apex, including peripheral lateral and para-median zone) were carried out by an urologist with six years of experience and more than 900 procedures performed. For each patient, two to four cores from the MRI-targeted areas were collected in addition to standard 12-core systematic sampling. An expert uropathologists, blind to the PI-RADS score, visually inspected the biopsy cores microscopically and assigned the Gleason score (GS).

### 2.4. Local Binary Pattern Features of Three Orthogonal Planes

To measure the tissue density distribution inside each ROI, we extracted statistical features exploiting the grey-level histogram and capturing the image intensity [10]. Twelve features were derived from the histogram of the 3D ROI for both T2 and ADC images: the mean, standard deviation, skewness, and kurtosis. The intensity distribution is also related to the histogram width, energy, entropy, the value of the histogram absolute maximum, the corresponding grey-level value, and the number of relative maxima in the histogram [11]. The three orthogonal planes local binary pattern (TOP-LBP) is a relatively new feature in radiomics [12,13]. The computation of a basic bidimensional LBP will now be explained. Considering a bidimensional image (I), 2D LBP compared the intensity (Ip) of each pixel (p) with the intensity (Ij) of all its j-th neighbor pixels that lay on a circle centered in p, with the radius ®. If Ij > Ip, the j-th pixel was set to 1, otherwise it was set to 0. Later, it was possible to process all p’s neighboring pixels in a circular direction, reading the sequence of 0 s and 1 s as a binary string and coding the value of p to the equivalent decimal value. By processing in this way all the pixels of I, it was possible to obtain a new image encoding the intensity distribution of each pixel in relation to its neighbors. This feature could capture part of the textural information of the original image. To extend a 2D LBP to the 3D environment, we introduced another 3D implementation of LBP transformation and considered the co-occurrence on three orthogonal planes crossing the center of the analyzed volume [14]. Furthermore, in our LBP implementation, we considered three more variants to cope with the other two issues of 2D LBP definition. First, we computed the rotation invariant LBP [15]; second, we implemented a uniform version of LBP; third, we combined together the two aforementioned variants, computing a rotation invariant and a uniform last variant. Finally, from the histograms calculated from the TOP-LBP and each of the variants, we extracted the same 12 statistical measures described before and concatenated the results into a 48-element vector. Applying this approach to the T2w and ADC projections, we obtained a final 96-feature vector.

### 2.5. 3D Gray Level Co-Occurrence Matrix Features

Co-occurrence matrix features represent how often a pixel with a specific greyscale intensity value occurs either horizontally, vertically, or diagonally to adjacent pixels with another intensity value [16]. This represents a measure of the tissue microstructure and can facilitate the prediction of PCa aggressiveness. Based on this consideration, we computed the 3D grey level co-occurrence matrix (GLCM3) for each region of interest (ROI), that is the 3D generalization of the bi-dimensional GLCM. Given a 3D grey-scale image (*I*) and a 3D Cartesian reference system (*O*) (*x*, *y*, *z*), whose origin is located in the top-front-left corner of *I*, the position of each voxel can be identified by a vector of **p** = p_x_*i* + p_y_*j* + p_z_*k*, with p_x_, p_y_, and p_z_ ∈ *N*. We also denoted a displacement vector of **d** = d_x_*i* + d_y_*j* + d_z_*k*, with d_x_, d_y_, and d_z_ ∈ *N*. If we define *m* as equal to the number of the bit used to represent *I*, a *GLCM3* is a square matrix of the size *N* = 2^m^, where each entry (*g_i_*, *g_j_*), with both *g_i_* and *g_j_* ∈ [0, 2^m^ − 1], represents the number of times a voxel in **p** with the intensity of *g_i_* is separated by a displacement (**d**) from another voxel with the intensity of *g_j_*, therefore located in **p** + **d**. Denoting as dh the h-th component of d, to assess all possible directions, we considered the combination of d c {−1, 0, 1} as displacements, without considering the (0, 0, 0) vector yielding to 26 different displacement directions. Finally, from each GLCM3, we extracted the autocorrelation, homogeneity, entropy, energy, covariance, inertia, and absolute contrast [17]. Then, concatenating such GLCM3 measures for T2 and ADC acquisitions, we obtained 36 × 7 × 2 = 364 GLCM3 descriptors.

### 2.6. Quantitative Analysis and Features Selection

**Figure 2:**
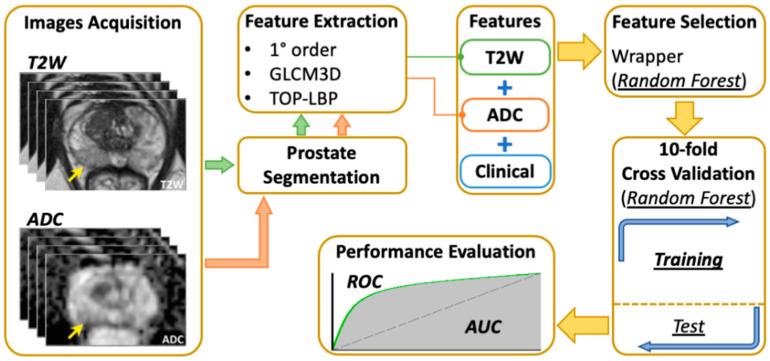
Machine-learning algorithm process.

All experiments were designed and executed within the Matlab 2017 developing environment and with the auxiliary help of the Weka 3.8.1 software. ROI was defined as total prostate volume. From ROIs, 484 image features from T2w and ADC were extracted using radiomic analysis as described before (24 statistical features–12 for each sequence– 364 GLCM3 descriptors, and 96 TOP-LBP). PCa aggressiveness was assessed by combining TOP-LBP, GLCM 3D, and the first order statistical features previously extracted. To empower our machine learning tool, we evaluated many semantic (clinical) features and selected the two most effective ones during radiomic analysis: the DRE of the prostate, and the highest PI-RADS score of the patient (PIRADSmax). These descriptors were combined with the previous image-based features. All the possible GS were collapsed into a binary pool so that the targets belonged to one of the two possible classes: targets with CS lesions containing PCa with a GS ≥ 7 (ISUP grade ≥ 2) and targets without CS PCa (GS < 7). The 487 features obtained formed the artificial intelligence (AI)-based model to predict the GS. In order to select the most effective features and to reduce the risk of low performance (because of a high number of redundant descriptors), a Wrapper feature selection was adopted, using a random forest algorithm with a bestfirst search [18,19] (Figure 2). The rationale of this classifier is that it can work with both qualitative and quantitative descriptors, even with a large data set, allowing us to perform, as a minimum, an internal algorithm validation, regardless of the number of ROIs. Practically, we tested several feature subsets, where each feature subset (*F_m_*) was composed of the first *m* features. We defined the best feature subset as follows: at the end of every internal cross-validation loop, we evaluated the classification performance obtained using *F_m_* and we compared them against the performance achieved using the other *F_m−1_*. If the performance was improved, the *m-th* feature added to the set was retained, and the internal loop was repeated using a new set *F_m+1_*, in which we added a new feature from those not used yet. If the performance was not improved, we kept on processing another feature. If no performance improvement was found for all the features not in *F_m−1_*, the search stopped, and *F_m−1_* was the best feature subset returned by the internal cross-validation loop.

Therefore, the diagnostic performance of each image feature was assessed by measuring the area under the curve (AUC) of the receiver operating characteristic (ROC) curve [20]. Diagnostic accuracy, sensitivity, and precision were determined on the best AUC obtained through our machine learning method. Accuracy, precision, and sensitivity were calculated using the best cut-off on each ROC, with 95% confidence intervals and a *p* value < 0.05, which is considered statistically significant.

## 3. Results

From the 135 patients included who underwent TRUS MRI target fusion biopsies plus 12-core systematic biopsies, 20 patients were excluded due to previous treatment for PCa; seven underwent a high-intensity focused ultrasound (HIFU) and 13 underwent cryotherapy. Remarkable image artefacts from previous orthopaedic procedures (hip replacement) determined the exclusion of 9 patients. Fifteen patients had precluded segmentation due to the size or location of their cancer lesions (tumors involving an entire lobe or the whole prostate gland). After this selection, only the 91 patients considered eligible underwent radiomic analysis (Figure 1). The median age of the patients (IQR) was 67 years (12). The meanean PSA (±standard deviation, SD) was 8.59 ng/mL (±6.29), and the mean prostate volume was 63.3 mL (±27.7). The most-represented PI-RADS score was 3, which was the score for45 patients, while the remaining 46 patients’ scores were were PI-RADS 4 and 5, in 27 and 19 patients, respectively. A total of 1371 prostate biopsy cores were collected, 279 of them from target cores. GSs 6 (3 + 3) and 7 (3 + 4) were the most representative of our population, for 20 and 17 patients, respectively. GS 7 (4 + 3) was found in 10 patients. No GS 10 was observed in our cohort. Thirty-two patients had a negative histology for PCa (Table 1) showing areas of benign hyperplasia and/or inflammation.

**Figure 3:**
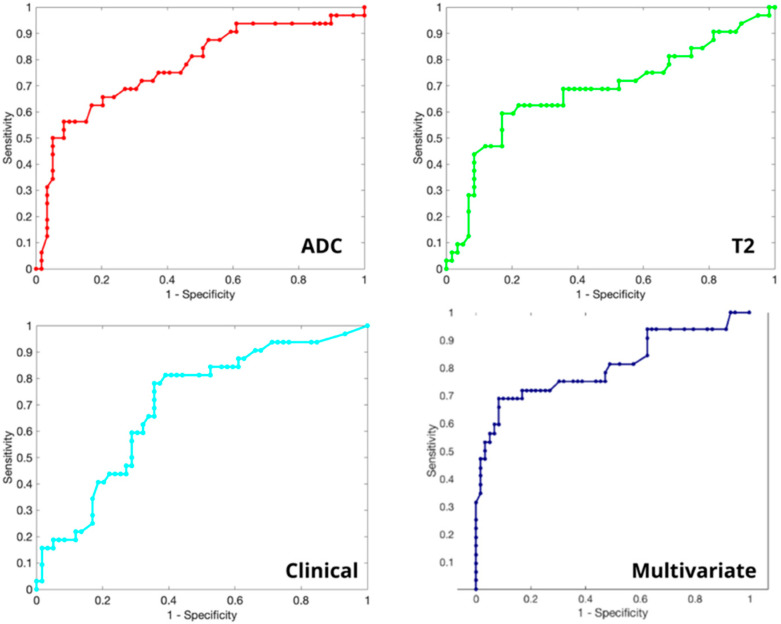
Receiver operating characteristic (ROC) curves of ADC WrapperRF (AUC = 0.774), T2 WrapperRF (AUC = 0.681), clinical (AUC = 0.698) and multivariate analysis (AUC = 0.804).

Of the 91 patients included in our cohort, we observed that 43% (n = 39) of ROIs belonged to those with CS PCa. At the end of the feature selection process, nine features were chosen and used to predict if GS was CS ≥ 7 in the final pathology through the classifier previously described (Figure 3). The features selected included a DRE and PIRADSmax, four being extracted from T2w images and three from ADC maps. In the univariate analysis (Table 2), the AUC values from the features extracted resulted to be lower than the AUC values from the combination of features.

Using the set of features detected by our cross-validation internal loop, the system achieved a detection accuracy for CS PCa equal to 83.5%, a CS precision of 84.4%, and a CS sensitivity of 91.5%. The resulting AUC of the multivariate analysis was 80.4% (Figure 3).

## 4. Discussion

Based only on mpMRI and PI-RADS scores, prostate biopsies may be useless, leading to a higher number of indolent tumors diagnosed, with major consequences on patients’ mental health and issues being raised with cancer overtreatment [21]. Even if PI-RADS improved the detection rate of CS PCa, it still has some limitations: it is reader-dependent, it does not give information about tumor aggressiveness, and it wasn not designed for 3D volume delineation. In this study, we have developed a radiomic-based machine learning model for attempting PCa aggressiveness prediction to overcome the PI-RADS score limits. Previous studies on GLCM-based textural features derived from ADC maps showed promising results in differentiating between benign and malignant tumors in the peripheral zone (PZ), and between GS 6 and GS ≥ 7 cancers [22,23]. In this study, we focused on T2w images, which are easy to acquire, less prone to artefacts, and suitable for every patient. T2w offers a high signal-to-noise ratio, a high spatial resolution, and soft tissue contrast images of the prostate gland, as important feature in PI-RADS score [24]. On the other hand, DWI sequences, and more specifically, ADC maps, thanks to the higher contrast resolution, are considered one of the most accurate and effective sequences for PCa assessment during MRI [25]. Rozenberg et al. combined ADC-derived features and logistic regression to predict the aggressiveness of PCa with an accuracy of 75% [26]. Another study, integrating features based on pharmacokinetic model parameter maps, showed an accuracy of 77% for detecting neoplasms in the PZ [27]. Our radiomics allowed us to elaborate on a tool that was able to predict CS PCa with an accuracy of 83.5%. Other radiomic analyses based on T2w and DWI or ADC MRI images have been reported in the literature. Chaddad et al. combined features derived from T2w and DWI to predict PCa invasiveness with an AUC of 65% [28]. In our results, the AUC was 80.4%, and we observed that not all radiomic features are equally effective in identifying PCa aggressiveness. By extracting the same number of features from both T2w and ADC acquisitions, almost 500 descriptors were analyzed. Hopefully, this provided robustness to the results and enabled the classifier to build a more generalized interpretation model of the data. Some authors have recently tried to develop and validate nomograms for the prediction of CS PCa. Nevertheless, these nomograms focused on a specific population with grey-zone PSA level (4–10 ng/mL) or PI-RADS 3 lesions [29,30]. Instead of targeting our work only on a pre-specified category, we avoided this and superselected our population in order to obtain data from a real-life cohort and a radiomic tool that could be more generalizable.

The classifier used was a random forest classifier due to its well-known ability to deal with a large amount of features [18]. To get this method working with the distinguishing features between the CS and the NCS PCa groups, a feature selection algorithm [19] was applied as a pre-processing step. Using a best-first wrapper algorithm, the amount of extracted features was reduced to a smaller pool of nine salient features. The rationale behind the choice of this classifier was that it can work with both qualitative and quantitative descriptors, even with a large data set. Using such an approach, we were able to consider all the features together for the selection, rather than focusing on each of them using a more limited univariate method. Both the over-fitting and challenge of dimensionality problems, typical of machine learning approaches, were tackled through two main choices: first, by using a random forest classifier, which is accredited as being less prone to overfitting and as more robust to the problems associated with an excessive number of features compared to the samples in the dataset; second, the feature selection phase was a strategy aimed at eliminating the least relevant measures which, in turn, reduced the risk of overfitting the data. The TOP-LBP overcame the intrinsic limitations of the planar approach, such as slice orientation dependency and slice gaps, making the analysis more flexible and able to capture the pattern of the segmented tissue along the volumetric shape. Finally, we selected four first-order statistical features, two clinical (semantic) features, and three other textural features. This combination of features made it possible to capture potential differences between CS and NCS PCa, providing results that seem to support the clinical benefit of MRI radiomic analysis for the prediction of PCa aggressiveness. This method appears to provide a non-invasive learning tool using mpMRI with a CS precision of 84.4% and a CS sensitivity of 91.5%. Moreover, while predicting PCa aggressiveness, we included non-cancerous observations (GS = 0), achieving an average AUC of 80.4%.

This study has the following limitations: first, it was a single-center data radiomic analysis and future works will need to be performed to get an external validation. Second, it did not distinguish between PZ and TZ PCa, in fact, we focused on PZ PCa assuming that TZ PCa, having low malignant potential, would be included in NCS PCa. Third, all pathological results were only biopsy-proven and lacked further confirmation with radical prostatectomy specimens. Fourth, 3D analysis was applied to MRI slice gaps. About this problem, we think that the 3D machine learning algorithm, which obtained the most important information just from the images, might compensate for the slice gaps. Moreover, the multivariate analysis would make a contribution to managing the anisotropy of the images.

## 5. Conclusions

Radiomic analysis in patients undergoing targeted fusion biopsies could improve the detection rate of CS PCa and overcome the limitations of the subjective interpretation of MRI images, reducing the number of useless biopsies and the side effects that go with them. Our machine learning tool showed a detection accuracy for CS PCa of 83.5%, with a CS precision of 84.4%, a CS sensitivity of 91.5%, and a resulting AUC of 80.4%. These results suggest that our radiomic approach might deserve to be developed in further studies. In other words, multicentric studies are needed to validate our radiomic machinelearning tool.

## Figures and Tables

**Table 1:** Patients’ characteristics.

Number of Patients	91
Age, median (IQR) (years)	67 (12)
PSA (mean ± SD) (ng/mL)	8.59 (±6.29)
PSA density (mean ± SD) (ng/mL/cc)	0.163 (±0.134)
Prostate volume (mean ± SD) (mL)	63.3 (±27.7)
PI-RADS 3	45
PI-RADS 4	27
PI-RADS 5	19
Total target cores	279
Total random cores	1092
Target cores, median (IQR)	3 (1)
Positive biopsies	9 (65%)
CS PCa	39 (43%)
NCS PCa CS PCa PI-RADS 3 CS PCa PI-RADS 4 CS PCa PI-RADS 5	20 (22%) 5 (12.8%) 11 (28.2%) 23 (59%)
GS 3 + 3	20 (22%)
GS 3 + 4	17 (19%)
GS 4 + 3	10 (11%)
GS 4 + 4	9 (10%)
GS 4 + 5	2 (2%)
GS 5 + 4	1 (1%)
Negative (non-cancerous) biopsies	32 (35%)

IQR: interquartile range; SD: standard deviation; CS: clinically significant; NCS: non clinically significant; PCa: prostate cancer; GS: gleason score.

**Table 2:** Features description and univariate Area Under the Curve (AUC).

Univariate AUC	Feature Name	Feature Description
0.659	DRE	digital rectal exploration
0.675	PIRADSmax	Maximum PI-RADS value assigned to the tissue’s sample
0.563	T2 Histogram—kurtosis	The amount of voxels in T2w acquisition that differs >2 standard deviations from its average value
0.426	T2 Histogram—number of maximum relatives	The number of relative maximums in the gray level distribution of a T2w acquisition
0.426	T2 Histogram—energy around maximum relatives	The homogeneity of the pattern around the relative maximums of a T2w acquisition
0.469	T2 Range—TOP-LBP rotation invariant	The difference between the higher and the lower value of the LBP transformation in a T2 acquisition (without considering their rotation)
0.461	ADC Histogram—skewness	The asymmetry in the gray level distribution of an ADC acquisition
0.469	ADC Range—TOP-LBP uniform rotation invariant	The difference between the higher and the lower value of the LBP transformation in an ADC acquisition without considering their rotation and noisy patterns
0.452	ADC Histogram—TOP-LBP uniform rotation invariant	The number of relative maximums in the distribution of the LBP transformation in an ADC acquisition not considering their rotation and noisy patterns

AUC: area under the curve; ADC: apparent diffusion coefficient; LBP-TOP: three orthogonal planes-local binary pattern; LBP: local binary pattern.

## Data Availability

Not applicable.
